# m6A demethylase FTO and osteoporosis: potential therapeutic interventions

**DOI:** 10.3389/fcell.2023.1275475

**Published:** 2023-11-10

**Authors:** Mei Huang, Jianmin Guo, Lifei Liu, Haiming Jin, Xi Chen, Jun Zou

**Affiliations:** ^1^ School of Kinesiology, Shanghai University of Sport, Shanghai, China; ^2^ Department of Rehabilitation, The People’s Hospital of Liaoning Province, Shenyang, China; ^3^ Department of Orthopaedics, The Second Affiliated Hospital and Yuying Children’s Hospital of Wenzhou Medical University, Wenzhou, China; ^4^ School of Sports Science, Wenzhou Medical University, Wenzhou, China

**Keywords:** FTO, m6A methylation, bone metabolism, FTO inhibitor, osteoporosis

## Abstract

Osteoporosis is a common bone disease, characterized by a descent in bone mass due to the dysregulation of bone homeostasis. Although different studies have identified an association between osteoporosis and epigenetic alterations in osteogenic genes, the mechanisms of osteoporosis remain unclear. N6-methyladenosine (m6A) modification is a methylated adenosine nucleotide, which regulates the translocation, exporting, translation, and decay of RNA. FTO is the first identified m6A demethylase, which eliminates m6A modifications from RNAs. Variation in *FTO* disturbs m6A methylation in RNAs to regulate cell proliferation, differentiation, and apoptosis. Besides, FTO as an obesity-associated gene, also affects osteogenesis by regulating adipogenesis. Pharmacological inhibition of FTO markedly altered bone mass, bone mineral density and the distribution of adipose tissue. Small molecules which modulate FTO function are potentially novel remedies to the treatment of osteoporosis by adjusting the m6A levels. This article reviews the roles of m6A demethylase FTO in regulating bone metabolism and osteoporosis.

## 1 Introduction

Bone is a highly adaptive and dynamic tissue in the body, mainly comprising cells of multiple lineages, type 1 collagen, and mineral. The integrity of the bone structure is maintained through continuously remodeling by osteoclasts and osteoblasts (Zaidi, 2007). Bone homeostasis is influenced by a variety of factors, with both general ageing and oestrogen deficiency causing imbalances in bone metabolism (Manolagas, 2010; Ceco et al., 2017). When bone homeostasis is dysregulated, the excessive bone loss causes osteoporosis (OP), which is characterized by bone microstructure degradation, decreased bone mineral density (BMD), and then increased risk of fracture (Siris et al., 2014). With the rapid development of the aging population, the incidence of OP is continually rising. More than 200 million people suffer from OP of different degrees worldwide (De Martinis et al., 2021). Fractures caused by OP have become a serious public health problem because of the associated high morbidity, disability, and mortality rates, which lead to the consumption of extensive social public health resources (Eastell et al., 2016; Ebeling et al., 2022).

Currently, m6A modification attracts great interest due to its important roles both in the various biological processes and pathogenesis of multitudinous diseases (Du et al., 2021). m6A modification, occurs in the adenosine base at the nitrogen-6 position of mRNAs and interferes with more than 7,000 mRNAs in mammalian transcriptomes (Dominissini et al., 2012; Zhou et al., 2016). Abnormal m6A modification is correlated with the proliferation, survival, and invasion of cancer cells (Zhang et al., 2016; Du et al., 2017; Cai et al., 2018; Chen et al., 2019; Lin et al., 2019).

**FIGURE 1:**
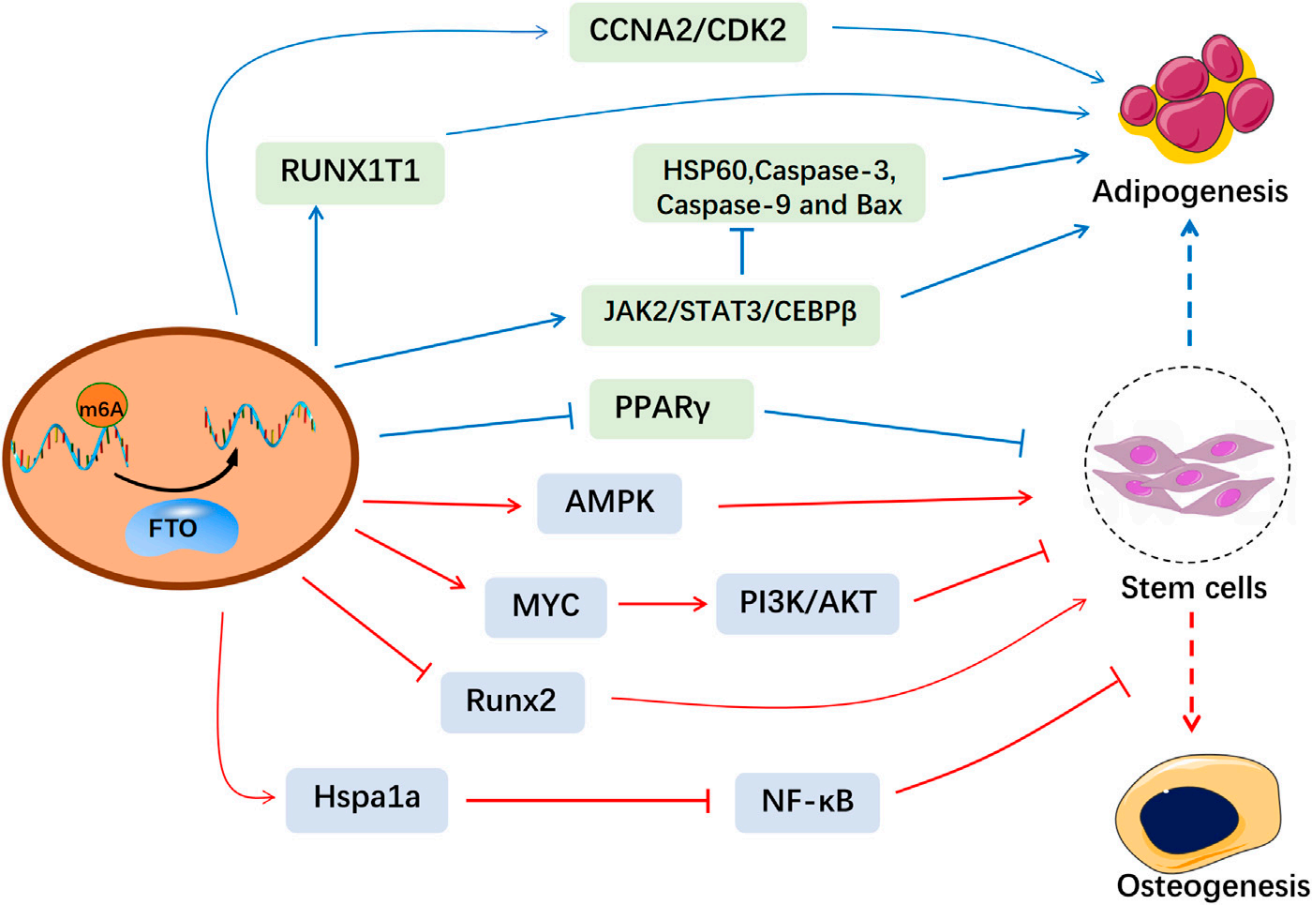
The molecular mechanism of FTO in the osteogenesis and adipogenesis of MSCs. FTO-mediated m6A modification affects the bone microenvironment and the biological functions of MSCs, which provides a therapeutical window for osteoporosis.

Fat mass and obesity-associated protein (FTO) is the first identified m6A demethylase, which eliminates m6A modifications from RNAs (Jia et al., 2011). FTO was initially reported strongly related to polygenic obesity in humans (Dina et al., 2007; Zhao et al., 2014a). Then FTO is found partially located on nuclear speckles and FTO depletion remarkably increases the total m6A levels (Jia et al., 2011). Variation in *FTO* affects m6A methylation and thus disturbs physiological activities (Wang et al., 2020; Qing et al., 2021). Recently, m6A methylation has been reported to regulate bone metabolism via modulation of hormones, cytokines (Huang et al., 2021a). Given the essential roles of epigenetics in regulating bone homeostasis, FTO-mediated m6A methylation has been found to be associated with bone-related diseases (Wang et al., 2021). In this review, we summarized the influence of FTO-mediated m6A methylation in bone formation and resorption (Figure 1), which helps to explore the pathogenesis of osteoporosis and provide therapeutic strategies based on epigenetics.

## 2 FTO biology

The FTO gene was initially reported as an obesity-susceptibility gene that predisposes individuals to obesity (Dina et al., 2007; Peeters et al., 2008; Price et al., 2008). *FTO* gene is located on human chromosome 16 and regulates energy balance by controlling energy consumption (Fischer et al., 2009). Human FTO is about 400 kb in length and consists of 8 introns and 9 exons, encoding a variety of protein products. The FTO gene has high homology in mammalian species such as pigs, mice and other mammals (Stratigopoulos et al., 2008). The FTO protein belongs to the Fe(II)- and 2-oxoglutarate-dependent dioxygenases family (Gerken et al., 2007). Structural analysis revealed two domains of FTO: an amino–terminal domain (NTD) and a carboxy–terminal domain (CTD). The NTD is the core region of FTO demethylation activity, mainly consisting of a β-fold. While the CTD is composed largely of an α-helix, interacting with NTD to keep the stability of the protein (Han et al., 2010).

FTO is found partly located on nuclear speckles and widely expressed in fetal and adult tissues (Jia et al., 2011). The expression level of *Fto* mRNA is the highest in white adipose tissue and specific brain regions across mouse tissues. Moderate levels of *Fto* expression were revealed in calvaria bones, bone marrow and femur, while low expression was observed in skeletal muscle and heart (Zhang et al., 2019). FTO is the first classified m6A demethylase, which performs methylation modification at the N6 position of adenosine (Dominissini et al., 2012; Meyer et al., 2012) and interferes with genome-wide m6A demethylation (Wei et al., 2018). It is the most widespread internal chemical modification affects over 7,000 mRNAs in mammalian transcriptomes (Zhou et al., 2016). The functions of m6A are regulated by the interplay of methyltransferases (“writers”), binding proteins (“readers”), and demethylases (“erasers”) (Panneerdoss et al., 2018; Shi et al., 2019). The m6A writers methyltransferase-like 3 (METTL3), methyltransferase-like 14 (METTL14), and Wilms’s tumor 1-associating protein (WTAP) transfer methyl groups from reactive methyl compounds onto the N-6 position of adenosine (Ping et al., 2014). The m6A erasers FTO and AlkB homolog 5 (ALKHB5) can reversibly detach the methyl groups of m6A off RNAs (Jia et al., 2011; Zheng et al., 2013). FTO can oxidize m6A to N6-hydroxymethyl adenosine (hm6A) as an oxidation intermediate through intermediate modification of 2-oxoglutarate-dependent dioxygenase and ferrous ions. Then FTO performs further oxidation on the metastable hm6A in the same way to form an unprecedented N6-formyladenosine (f6A). Under physiological conditions, hm6A and f6A have a half-life of ∼3 h in an aqueous solution and then finally decompose into adenine (A) (Fu et al., 2013). After FTO removes the methyl group of m6A, the original sites of m6A methylation cannot be identified by “reader” proteins, which affects multiple physiological and pathological processes (Li et al., 2022; Yang et al., 2022). Abnormal expression and function of FTO are related to the occurrence of bone metabolic disorders. Therefore, this review mainly focuses on the role of FTO in bone metabolism and its regulatory mechanism.

## 3 Impact of FTO on osteogenesis

Bone metabolism is a dynamic process of bone formation and resorption, in which multiple growth factors and signaling pathways are implicated (Clezardin et al., 2021). Osteogenesis that begins from bone marrow mesenchymal stem cells (BMSCs) to osteoblasts is extremely important in skeleton formation and regeneration. The complex correlation of m6A modification with osteogenesis has been consecutively explored. Growing evidence had indicated that FTO-mediated m6A modification is a novel regulator in regulating bone cells (Table 1).

**TABLE 1:** Regulation roles of FTO-mediated m6A Demethylation in bone development and bone homeostasis.

Regulators	Species and cells	Biological function of FTO in bone development	Target genes or pathways	Refs
FTO	Mouse (KO)	Maintain BMD and BMC	-- --	Sachse et al. (2018)
		↑ALP of Plasma biochemistry		
FTO	Mouse (inhibitor-- IOX3)	Maintain BMD and BMC	-- --	McMurray et al. (2015)
FTO	OP patients	↓osteogenic differentiation	↓Runx2	Wang et al. (2021)
	Human BMSCs(shRNA)			
	OVX Mouse			
FTO	Human BMSCs(siRNA)	↓osteogenic differentiation	↑MYC/PI3K/AKT	Zhang et al. (2020a)
	OVX Mouse			
FTO YTHDF1	Patients with osteonecrosis	↑osteogenesis	↓PPARγ	Chen et al. (2022)
	Human MSCs(shRNA&inhibitor)			
	Mouse(KO)			
FTO	C3H10T1/2 cells (OE and siRNA)	↑osteogenic differentiation	↑AMPK	Son et al. (2020)
			↑Dlx5 and ↑Runx2	
FTO	Mouse (KO)	↑Protect osteoblasts from genotoxic damage	↑Hspa1a	Zhang et al. (2019)
	Osteoblast		↓NF-κB	
FTO	Mouse and porcine preadipocytes (OE and siRNA)	↑adipocyte differentiation	↑JAK2/STAT3/CEBPβ	Wu et al. (2019)
FTO	3T3-L1 cells (siRNA)	↓adipocyte apoptosis	↑JAK2/STAT3	Shen et al. (2021)
	Mouse (FTO injection)		↓HSP60, Caspase-3, Caspase-9 and Bax	
FTO	Human preadipocytes(siRNA)	↑adipogenesis	↑RUNX1T1	Zhao et al. (2014a)
			↓SRSF2	
FTO YTHDF2	3T3-L1 preadipocytes(siRNA)	↑adipogenesis	↑CCNA2 and ↑CDK2	Wu et al. (2018a)
FTO YTHDF2	Porcine primary preadipocytes	↑adipogenesis	↑CCNA2 and ↑CDK2	Wu et al. (2018b)
	Mouse 3T3-L1 preadipocytes (siRNA and OE)			
FTO	RAW264.7 cells and BMMs(shRNA)	↑osteoclastogenesis	↑NF-κB	Zhuang et al. (2021)
			↑NFATc1	

### 3.1 FTO affects osteogenic differentiation of BMSCs

BMSCs possess the ability of highly self-renewal and multilineage differentiation, which are potential cellular sources for bone formation. Keeping a coordinated stem cell differentiation program is essential for bone development. The aberrant expression of FTO in MSCs from different bone diseases further supported its roles in osteogenesis. The level of methyltransferase FTO is increased in BMSCs from ovariectomy (OVX) mouse and OP patients with the decrease of m6A methylated RNA level (Wang et al., 2021). Overexpression of FTO in BMSC promoted the progression of osteoporosis by inhibiting osteogenic differentiation through demethylation of Runx2 gene, and inhibition of FTO in ovariectomized mice showed increased bone formation, partially alleviating OVX-induced osteoporosis. From this study, FTO appears to play a bad role in the development of osteoporosis (Wang et al., 2021).

However, another study showed that FTO was obviously upregulated during the osteogenic differentiation of human MSCs and markedly declined in patients with osteonecrosis. Knockdown of FTO via either shRNA or utilization of two FTO inhibitors FB23 or FB23-2 diminished the osteogenesis of human MSCs. Consistently, conditional knockout of Fto in mice bone was accompanied by the reduction of bone mineral density and damaged bone regeneration (Chen et al., 2022). Son et al. found FTO expression also significantly increased in C3H10T1/2 cells and promoted osteogenic differentiation after BMP2 treatment (Son et al., 2020). Mechanically, FTO induced mild endoplasmic reticulum (ER) stress through a positive feedback loop with p-AMPK and upregulating the osteogenic genes Distal-less homeobox5 (Dlx5). Whereas severe ER stress attenuated FTO expression and AMPK activation of C3H10T1/2 cells, thereby inhibiting osteogenic differentiation (Son et al., 2020). Besides, MicroRNAs (miRNAs) also regulate the differentiation of BMSCs into specific lineage by targeting FTO. miR-149-3p increased the extracellular matrix maturation and mineralization in BMSCs through directly inhibiting FTO (Li et al., 2019). miR-22-3p delivered by BMSC-isolated Extracellular vesicles (EVs) could negatively target FTO, repressing the MYC/PI3K/AKT pathway, thus promoting osteogenic differentiation *in vivo* and *in vitro* (Zhang et al., 2020a).

The effects of FTO on osteogenic differentiation of BMSCs in the above findings are controversial, but such results may not be contradictory. The first thing that is clear to us is that FTO indeed regulates the biological function and cell fate of BMSCs through osteogenic genes and signaling pathways involved in the regulation of osteogenesis (Plotkin and Bellido, 2016; Lowery and Rosen, 2018). Secondly, we speculated that FTO has different effects on osteogenic differentiation in cellular stress (such as disease) and normal physiological states. Meanwhile, the expression level of FTO is critical to the effect of osteogenic differentiation of cells. From the above experiments, it was found that knockdown or overexpression of FTO at the cellular level resulted in a reduction in osteogenic differentiation. Inhibition of FTO in ovariectomized mice with high FTO expression promoted osteogenesis and alleviated osteoporosis. Thus, a certain level of FTO may need to be maintained in the skeleton to facilitate bone formation.

### 3.2 FTO affects adipogenic differentiation of BMSCs

On the flip side, adipogenesis of BMSCs is also an essential part of bone homeostasis (Tencerova et al., 2019). Adipose tissue negatively regulates osteogenesis through the secretion of adipokines like adiponectin and omentin (de Paula and Rosen, 2020). FTO, a well-known obesity gene, has also been found to influence skeletal development by regulating adipogenesis in the bone marrow. FTO accelerates adipogenesis in BMSCs by demethylating mRNA for Peroxisome proliferator-activated receptor gamma (Pparγ) (Shen et al., 2018). Pparγ, a critical regulator of adipogenesis, was also identified as a biomarker for osteoporosis. Likewise, FTO modulates alternative splicing of adipogenesis-related factor runt-related transcription factor 1 (RUNX1T1) to control preadipocyte differentiation in an m6A-dependent manner (Zhao et al., 2014b). Consistently, FTO deficiency hindered adipogenesis of preadipocytes through JAK2-STAT3 signaling. Mechanistically, Loss of FTO reduced JAK2 expression and STAT3 phosphorylation in an m6A-dependent manner, thereby attenuating transcription of C/EBPβ, which blocked the early stage of adipocyte differentiation (Wu et al., 2019). On the other hand, FTO also inhibited adipocyte apoptosis through triggering JAK2/STAT3 signaling pathway and weakening Hsp60 mRNA m6A modification to block mitochondrial unfolded protein response (Shen et al., 2021).

Moreover, FTO also affects the cell cycle. Wu et al. demonstrated that two key cell cycle regulators, cyclin A2 (CCNA2) and cyclin-dependent kinase 2 (CDK2), can be demethylated and enhanced in expression by FTO. Subsequently, YTHDF2 distinguishes and destabilizes m6A-modified CDK2 and CCNA2 mRNA. FTO deficiency caused the downregulation of CCNA2 and CDK2 expression, which in turn blocked the cell cycle and suppressed the adipogenesis of BMSCs. FTO/m6A/YTHDF2 signaling plays an important role in regulating cell cycle and adipogenesis of preadipocytes (Wu et al., 2018a). Likewise, another study also showed targeting inhibition of FTO via Epigallocatechin gallate (EGCG) reduced m6A-dependent CCNA2 and CDK2 expressions and restrained adipogenesis in the m6A-YTHDF2 dependent mechanism (Wu et al., 2018b).

Consistent with the results of the cellular experiments described above, Fto-deficient mice displayed a significant reduction in adipose tissue (Fischer et al., 2009; Ronkainen et al., 2016). However, while osteoporosis is partly due to increased adipose differentiation and decreased osteogenic differentiation of BMSC, leading to a decline in overall bone mass. Excessive suppression of bone marrow adipogenesis may be detrimental to bone development. FTO demethylase activity also plays an indispensable role in normal bone growth and mineralization. The deficiency of FTO enzymatic activity leads to a marked reduction of BMD and bone mineral content (BMC), similar to that seen in osteoporosis. The mechanism behind the reduction of BMD and BMC remains to be clarified, but the decrease in alkaline phosphatase (ALP) level indicates that osteoblast function may be affected (Sachse et al., 2018). This may be due to the ability of bone marrow adipocyte to store and provide energy, which can affect neighboring bone cells (de Paula and Rosen, 2020). Some studies have proposed that the marrow adipocyte may be an osteoblast with a large lipid droplet. During early differentiation, osteoblasts contain small lipid droplets which are rapidly lipolyzed in response to energy requirements (Robles et al., 2019). Certain lineage tracing studies indicated that some adipocytes also have labeling characteristics of osteoblasts (McGee-Lawrence et al., 2016). In summary, FTO is an important regulator of BMSC fate and further exploration will provide new insights into the intercellular transformation in bone marrow.

### 3.3 Impact of FTO on osteoblasts

Osteoblasts are differentiated from BMSCs and transform into osteocytes located in the mineralized bone matrix. The proliferation of osteoblasts plays a vital role in the maturation and mineralization of the bone matrix. Osteoblast-mediated bone formation is essential in maintaining bone mass and strength. Fto-knockout mice decreased the activity of osteoblasts *in vivo* (Chen et al., 2022).

Interestingly, mice conditionally knockout Fto in osteoblasts (*Fto* ^*Oc*^ ^KO^) exhibited normal growth but showed age-related bone volume reduction. 12-week-old *Fto* ^Oc KO^ mice showed no obvious alterations in bone mass compared to wild-type mice but had lower bone volume than wild-type mice at 30 weeks of age. The decrease of osteoblast/osteocytes activity in *Fto* ^Oc KO^ mice resulted in the reduction of bone volume, which led to low bone turnover and accumulation of marrow fat. Fto KO osteoblasts were more vulnerable to genotoxic substances (H2O2 and UV), increasing the sensitivity of osteoblasts to cell death and promoting apoptosis. Meanwhile, increased expression of heat shock protein family A (Hsp70) member 1A (Hspa1a) or inhibition of nuclear factor-kappa B (NF-κB) signaling largely restored increased apoptosis and the defective mineralization of FTO deficient osteoblasts. Thus, FTO regulated osteoblast apoptosis via the Hspa1a–NF-κB pathway (Zhang et al., 2019). However, Shen et al. found conditional Fto knockout in osteoblasts repressed bone loss in OVX mice but not in sham-operated group. FTO was upregulated in the process of aging and osteoporosis in mice, which promoted the shift of BMSCs to adipocytes rather than osteoblasts and inhibits bone formation through demethylating the mRNA of PPARγ (Shen et al., 2018). These differences indicate that FTO in different animal models may have separate functions during osteopenia. FTO needs to be kept at a certain level to maintain bone mass. Low or high FTO expression is not conducive to bone formation. Further studies on molecular mechanisms are still needed.

## 4 Impact of FTO on osteoclasts

Osteoclasts, bone-resorbing cells, which originate from myeloid precursors, undergo several stages of differentiation (Jacome-Galarza et al., 2019). Under physiological conditions, bone remodeling consists of osteoblast-mediated bone formation and osteoclast-mediated bone resorption. The pathological cause of osteoporosis is a disturbance in these two balanced processes (Durdan et al., 2022). A study indicated FTO expression was upregulated in bone marrow monocytes (BMMs) from OVX mice. FTO overexpression facilitated RANKL-induced binding of NF-κB to NFATc1 promoter and then promoted osteoclast differentiation in RAW264.7 and BMMs cells. FTO knockdown through periosteal injection of lentiviral shRNA-FTO inhibited osteoclastogenesis and bone resorption in osteoporotic mice (Zhuang et al., 2021). NF-κB, a downstream factor of RANKL/RANK signaling pathway, is essential in mediating the differentiation and maturation of osteoclasts (Park et al., 2017). Another research also showed that FTO depletion restrained the NF-κB signaling pathway via reducing the phosphorylation levels of p65, IKKα/β and IκBα in RAW264.7 cells and bone-marrow-derived macrophages (Gu et al., 2020). In addition, silencing FTO also markedly raised the expressions of pro-inflammatory markers (TNF-α and iNOS) in macrophages (Hu et al., 2019). Due to its important roles in osteoclast-specific genes, FTO is a promising therapeutic target on osteoporosis caused by abnormal bone resorption.

## 5 Small-molecule inhibitors targeting FTO demethylation activity

FTO is functionally essential in physiological processes, and its dysfunction has been related to various human diseases. Therefore, exploiting small-molecule modulators to utilize their biological function and excavate the pathogenic mechanisms are warranted. Flavonoids are polyphenols with high polarity and poor water solubility, which are difficult to be absorbed. Flavonoids can eliminate free radicals and play the role as antioxidants (Hernandez et al., 2009; Zhang et al., 2020b). Several studies have reported the role of flavonoids in improving bone health due to their anti-inflammatory and antioxidant properties (Preethi Soundarya et al., 2018; Ramesh et al., 2021). Flavonoids contain hydroxyl groups which readily form hydrogen bonds with amino acid residues, whereas low polarity compounds tend to bind to hydrophobic residues of FTO proteins. Chen et al. found the natural product Rhein has strong interaction with FTO. Rhein is a naturally existing anthraquinone with multifarious pharmacological effect (Cheng et al., 2021), which has been classified to bind with FTO and abolishes its demethylase activity (Chen et al., 2012). Rhein regulates m6A methylome rearrangement and represses adipogenic differentiation in a dose-dependent manner (Huang et al., 2021b). But Rhein can concurrently combine with FTO or alkB (Li et al., 2016). Implementing a method for selective restraint of FTO over ALKBH5 remains important. Besides, Rhein and its derivatives have been reported to have anti-osteoclastogenesis activity, inhibiting the TRAP activity and osteoclast differentiation (Xu et al., 2016; Jiang et al., 2019).

**FIGURE 2:**
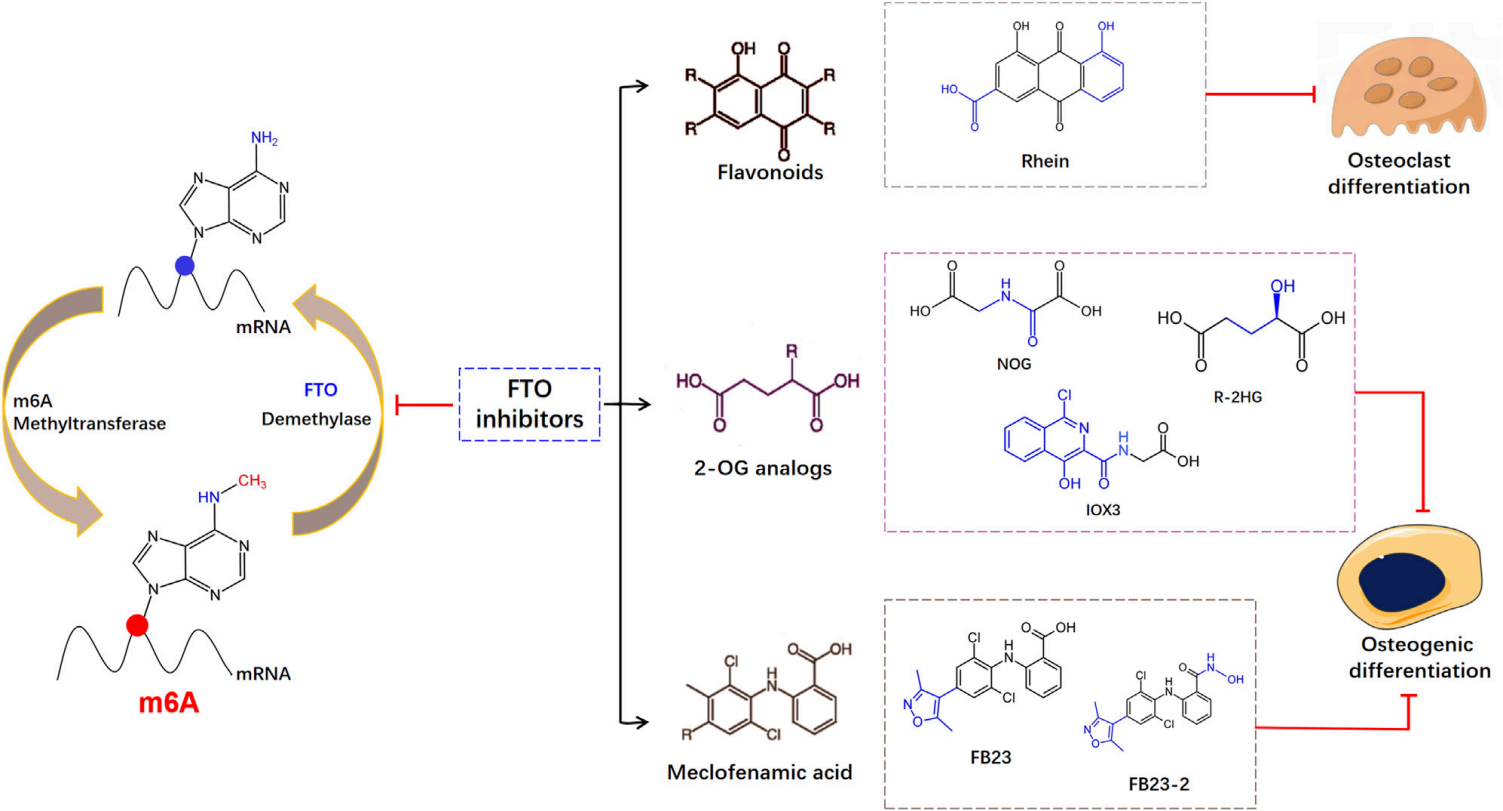
Schematic diagram of the structures of different inhibitors on FTO during the reversible m6A modification.

The FTO protein contains two-domain of 2-OG- and Fe(II)-dependent dioxygenases, showing -methyladenosine demethylase activity. A set of diverse 2-oxoglutarate (2-OG) analogs are selective inhibition of FTO. Studies have found that mice treated with IOX3, an effective inhibitor of FTO demethylase activity, showed significant reductions in BMD and BMC (McMurray et al., 2015). But IOX3 was also an inhibitor of other 2OG oxygenases, compounds designed to selectively inhibit FTO demethylase activity still needed further exploration. Aik et al. (2013) showed sodium oxalate restrained the FTO expression, and distinguished two broad-spectrum 2-OG oxygenase inhibitors: pyridine-2,4-dicarboxylate and NOG. Then FTO inhibitor R-enantiomer of 2-hydroxyglutarate (R-2HG) was found based upon the structure of NOG, which is a tumor metabolite generated by mutant isocitrate dehydrogenase (Su et al., 2018). The R-2HG dysregulated the osteogenic differentiation of MSCs via reducing the expression of early (IBSP and LPL) and late (BGLAP and Osterix) osteoblast differentiation-related genes (Liu et al., 2021). The oncometabolite R-2HG in AML cells stimulated epigenetic alteration and halted hematopoietic differentiation. Mechanically, R-2HG upregulated NF-κB-dependent genes in BMSC which promoted chemoresistance and proliferation of AML Cells (Chen et al., 2016). Meclofenamic acid (MA) is an anti-inflammatory drug that has been discovered as a selective inhibitor of FTO, which contends with FTO for the binding to the m6A-containing nucleic acid (Huang et al., 2015). Based on the structure of MA, the application of two MA-derived inhibitors, FB23 and FB23-2, impaired the osteogenesis of human MSCs (Chen et al., 2022). As shown in Figure 2, FTO inhibitors are classified according to their different structures. The binding sites and binding capacities of FTO inhibitors are different due to diverse structures. Given the functional role of FTO in the regulation of skeletal development and bone homeostasis, small molecule compounds targeting FTO can be developed to treat osteoporosis under the precise control of m6A modifications.

## 6 Conclusion and prospects

FTO-mediated m6A methylation is emerging as a novel target of bone development and bone metabolism. But the current comprehension of FTO-mediated m6A methylation in bone metabolism is still restricted. First, m6A methylation is a dynamic process, which requires multiple m6A methylation regulatory factors to work together in coordination. How the m6A methylation regulator coordinates and functions need to be further explored. Second, the molecular basis of FTO-mediated m6A methylation in bone resorption is not well elucidated. The effect of FTO on bone formation in different mouse models is still controversial. As discussed above, knockdown or overexpression of FTO in BMSC leads to a reduction in osteogenic differentiation. Inhibition of FTO in OVX mice promoted bone formation and alleviated osteoporosis. But FTO knockout prevented the skeleton from maintaining normal BMD and BMC. Thus, FTO may play a dual role in bone metabolism, requiring a certain level to maintain bone homeostasis, and the critical point of this process is not completely clear. And the role of FTO in osteoporosis in the current study is mainly focused on animal experiments, with fewer research in the population with osteoporosis. From the animal studies, it may be a potential biomarker for osteoporosis, but it needs to be further validated in clinical research on a larger population. Furthermore, small-molecule inhibitors of FTO have been identified and helped to understand its biological functions in multiple biological processes. Therefore, combining the precise regulation of m6A methylation in bone metabolism with small molecule inhibitors targeting FTO will provide new perspectives and treatment options for osteoporosis.
